# Values and value conflicts in implementation and use of preconception expanded carrier screening - an expert interview study

**DOI:** 10.1186/s12910-019-0362-1

**Published:** 2019-04-23

**Authors:** Amal Matar, Mats G. Hansson, Anna T. Höglund

**Affiliations:** Centre for Research Ethics and Bioethics, Department of Public Health and Caring Sciences, Uppsala University, Box 564, SE-751 22 Uppsala, Sweden

**Keywords:** Moral values, Intrinsic and extrinsic values, Preconception expanded carrier screening, Respect for persons, Solidarity, Human dignity, Health technology

## Abstract

**Background:**

Endeavors have been made to found and incorporate ethical values in most aspects of healthcare, including health technology assessment. Health technologies and their assessment are value-laden and could trigger problems with dissemination if they contradict societal norms. Per WHO definition, preconception expanded carrier screening is a new health technology that warrants assessment. It is a genetic test offered to couples who have no known risk of recessive genetic diseases and are interested pregnancy. A test may screen for carrier status of several autosomal recessive diseases and X-linked at one go. The technique has been piloted in the Netherlands and is discussed in other countries. The aim of the study was to examine values and value conflicts that healthcare experts recounted in relation to the discussion of implementation and use of preconception ECS in Sweden.

**Methods:**

We interviewed ten experts, who were associated with influencing health policymaking in Sweden. We employed systematizing expert interviews, which endeavor to access experts’ specialist knowledge. There were four female and six male informants, of which four were physicians, three bioethicists, one a legal expert, one a theologian and one a political party representative in the parliament. The participants functioned as members of two non-governmental bodies and three governmental organizations. We employed thematic analysis to identify themes, categories and subcategories.

**Results:**

Two main themes surfaced: values and value conflicts. The main categories of *Respect for persons, Solidarity, Human dignity, Do no harm, Health* and *Love* formed the first theme, while values conflicting with autonomy and integrity respectively, constituted the second theme. Concepts relating to respect for persons were the most commonly mentioned among the participants, followed by notions alluding to solidarity. Furthermore, respondents discussed values conflicting with Swedish healthcare ones such as equality and solidarity.

**Conclusions:**

The experts highlighted values and concepts that are distinctive of welfare states such as Sweden and delineated how preconception ECS could challenge such values. Moreover, the analysis revealed that certain values were deemed more substantive than others, judging by the extent and detail of inference; for example, respect for persons and solidarity were on top of the list.

## Background

Value theory, together with theories of rights, are the main branches of ethical theory [1]. While the former judges whether a state of affairs is good or bad, and as such is evaluative in nature, the latter is concerned with appraising actions as either morally right or morally wrong [1, 2]. Values have been categorized into either intrinsic or extrinsic values. An intrinsic value has been described as whatever is “valuable for its own sake, in itself, on its own, in its own right or as an end” [3]. An extrinsic value has been described simply as whatever that is non-intrinsic or as what is valuable for the sake of something it is connected to. Examples of intrinsic values include, freedom, love, health [4].

For decades now, endeavors have been made to found and incorporate ethical values and principles in most, if not all, aspects of healthcare. The prevailing moral rationales prompting activities related to healthcare are preserving lives and reduce suffering of those in ill-health, whether mentally or physically [5]. Furthermore, moral principles have been integrated into policymaking [6], communication [7], medical research [8] and health technology assessment [9]. As a result, numerous ethical guidelines and regulations stemmed from these ventures, for example; Declaration of Helsinki for Human Subject Research, issued by the World Medical Association [10].

In the United States of America, Beauchamp and Childress [11] introduced four principles for biomedical ethics. They maintained that the principles of autonomy, beneficence, non-maleficence and justice are universal and relevant in medicine, bioscience and healthcare. Across the Atlantic, Europe had different views. Commentators stated that for healthcare provision and medicine, these principles are lacking crucial values, such as care and compassion [12].

As a result, the European Commission sponsored a study plan in the 1990s, where researchers from different European countries identified four principles for ethical decision making in bioethics and bio-laws, namely; dignity, vulnerability, autonomy and integrity [13]. Human dignity has been emphasized and prized over autonomy, particularly for situations where autonomy is compromised, for example with comatose patients [12]. In 2006, the European Union issued a statement on the founding of common moral values for the European healthcare systems which were: universality, equity, access to good quality care and solidarity. In addition, the document detailed principles that are functional within the health system, such as ensuring patients’ safety and involvement, provision of quality and evidence-based care, and valuing redress and privacy/confidentiality of citizens [14].

In the Nordic countries, values and principles have instituted priority setting measures in healthcare. For example, in Sweden, priority setting procedure was founded on three values; human dignity, solidarity and medical needs, and cost effectiveness, where the hierarchy of the principles are significant in reaching a priority setting decision [15]. Maintaining the ranking of the principles is crucial in order to circumvent discrimination on the basis of age, personal choices/lifestyles or expensive treatments [16]. In fact, the cost effectiveness principle is only applied in appraisal of offers of treatment for the same disease [17].

Moreover, the Swedish Agency for Health Technology Assessment (HTA) - SBU, designed a framework to assess, among other things, ethical concerns of new technologies before their adoption by the healthcare system. Incorporated within this scheme are ethical principles of justice and equality, autonomy, privacy and cost effectiveness [18]. Furthermore, commentators agreed that assessing moral values as part HTA is essential because some HTs can bear moral sequels or/and the HT themselves possess moral values that may threaten the norms in a society. Lastly, they concurred that HTA itself is value laden because the process exhibits many value judgements such as deciding on which technology to evaluate, the type of evidence to use and the explication of research results [19]. Addressing values in HTA and HT can overcome the “dissemination problem”, and encourage uptake of efficacious HT by policymakers [5].

Preconception expanded carrier screening (ECS) is a new HT as defined by WHO [20], which involves a genetic test panel offered to a general population or to couples with no prior risk of recessive genetic diseases and are interested in becoming parents. A test screens for carrier status of several autosomal recessive and X-linked diseases at one go [21]. The technique has been piloted in the Netherlands [22] and was discussed in other countries [23–25]. To our knowledge there are no offers of preconception ECS in Sweden [26].

Since values and ethical principles are prominently considered in the discourse of healthcare, we found it pertinent to examine what values have been attended to in association with preconception expanded carrier screening (ECS) implementation and use in Sweden. Moreover, identifying the values and value conflicts can provide insights as to ways to improve dissemination of the new technology if it were to be implemented in Sweden.

### Aim

The aim of the study was to investigate values that experts described in relation to implementation and use of preconception ECS. The following research questions were examined:

- What values do experts express in relation to preconception ECS?
- Do experts assign different weights to the different recounted values?
- Do experts detail value conflicts between the described values? If so, what value conflicts are highlighted in relation to preconception ECS?
- According to experts, do any of the values associated with preconception ECS challenge societal norms in Sweden?

## Methods

The current study is a secondary analysis of the interview transcripts, which ensued from a previous study that examined Swedish policymakers’ views on ethical and social issues pertinent to preconception ECS use and implementation [27]. The present article concentrated on the informants’ discussion of values and value conflicts in relation to preconception ECS.

### Expert interviews

The transcripts were obtained by conducting expert interview with members of ethics committees in government and non-governmental organizations, that are associated with influencing health policymaking in Sweden. Expert interview gathers data from crystallizing points of knowledge and competency and thus has been viewed as a rapid and a good source of data. To select an expert, we adopted a social representational approach, which rely on expert’s status and their accomplishments. We performed systematizing expert interviews, as described by Bogner and Menz [28], which endeavor to access experts’ specialist area of proficiency in a certain field. The goal was primarily to access expert’s interpretative knowledge; in the form of their views, interpretations and reasoning. This was particularly important while carrying out secondary analysis of the transcripts. The method is described in further details elsewhere [27].

### Participants

The total number of participants were ten, with four female and six male informants. There were four physicians, three bioethicists, one legal expert, one theologian and one political party representative in the parliament. The participants functioned as members of national (nine respondents) and regional (one respondent) committees, or two non-governmental bodies and three governmental organizations, that attended to ethical and social issues pertaining to healthcare matters. Therefore, the included informants influenced health policymaking in Sweden.

The participants were working for or worked on board the following committees during the duration of data collection:

- SBU - Statens beredning för medicinsk och social utvärdering (The Swedish Council on Technology Assessment in Healthcare)
- SMER - Statens medicinsk-etiska råd (The Swedish Medical Ethics Council)
- The ethical board of the National Board for Health and Welfare - Socialstyrelsen
- Sveriges läkarförbund (The Swedish Medical Association)
- Svenska Läkaresällskapet (The Swedish Society of Medicine)

### Data collection

The duration of data collection lasted almost 10 months (from February till November, 2017). First, we consulted the websites of the previously mentioned committees to acquire contact information of potential participants and thereafter, we turned to a snowballing approach to recruit interview candidates. That means that we enquired of the respondents who were interviewed for contact details of potentially interested members in the aforementioned boards. A total of 30 members were approached and ten agreed to participate in our study. Reasons for declining were primarily lack of time, but some also expressed feelings of uncertainty about the topic (preconception ECS).

After a literature review, a semi structured questionnaire was drafted, appraised and accepted by all three authors. The questionnaire contained three major components; inquiries on participants’ background and function, questions on healthcare decision-making and, lastly, questions on ethical and social matters in relation to preconception ECS. The first author carried out the data collection and the interviews were conducted in English.

Most of the interviews (six) were concluded in more than an hour, while the time span was from 41 min to 68 min. Audio recordings of all interviews were performed and they were transcribed verbatim.

### Analysis

The transcripts were read a couple of times before a preliminary open coding was conducted, where we identified the texts conducive to moral values and principles. The analysis process was iterative to identify themes, categories and subcategories. The respondents referred to values such as autonomy, integrity, solidarity, human dignity, love which made up the categories and subcategories. The subcategories for the overarching categories of respect for persons, solidarity and human dignity were deductively inspired by literature. For example; Emanuel, Wendler [29] and Johnsson and Eriksson [30] formed the basis for respects for persons subcategories, Prainsack and Buyx [31] for solidarity and lastly Caulfield and Brownsword [32] for human dignity. However, we did not confine the analysis to values that were discussed in the literature, we were receptive to other categories as they transpired from the interview transcripts.

The analysis, using NVivo 11.4.3 software, was performed by the first author (AM). ATH analyzed three interviews independently and results were compared to analysis of AM. After discussion, differences were reconciled and agreement reached [33]. We followed thematic analysis method as described by Ryan & Bernard [34].

We ensured aspects of trustworthiness; credibility dependability and transferability were heeded while we planned the study, and collected and analyzed the data. Credibility was attained by opting for various genders, vocations, different committees; governmental and non-governmental, regional and national. We employed the method of expert interviewing to obtain specialized knowledge and experience of participants.

The duration of data collection was approximately ten months, which is relatively a short period during which there were minimal alterations to the interview guide. Moreover, all authors met several times during the analysis process to deliberate results and reach consensus. These measures were undertaken to fulfil the second feature of trustworthiness: dependability [33].

To achieve transferability, the third ingredient of trustworthiness, [33] we described at length, elsewhere, the health policymaking scene in Sweden, the roles of the boards where respondents belong and the methods used for selection and analysis of the data [27].

### Ethical considerations

According to Swedish Ethical Review Act, the study did not necessitate ethical review because it has not involved participants’ personal sensitive information. However, the authors followed the requirements for ethical conduct of research as described by Swedish law (SFS 2003: 460) [35].

An e-mail invitation of participation in our study was sent to all potential candidates, attached to which was an overview of the project and background information on preconception ECS. Upon approval of the participant, a time and a location where privacy could be maintained were agreed upon. We requested the consent from all participants to record our interviews and the audio files, together with the transcripts where their names were replaced with a code, were password-secured on a computer. Only the authors had access to the data. Moreover, in the e-mail request, we notified the research subjects of their capacity to withdraw from the study anytime they wished with no explanation as to why.

## Results

The analysis of the transcripts elicited two main themes; values and value conflicts. The first theme gave rise to six categories namely, *respect for persons, solidarity, human dignity, do no harm, health and love.* Several subcategories emerged for *respect for persons, solidarity, do no harm and human dignity categories*.

**Fig. 1:**
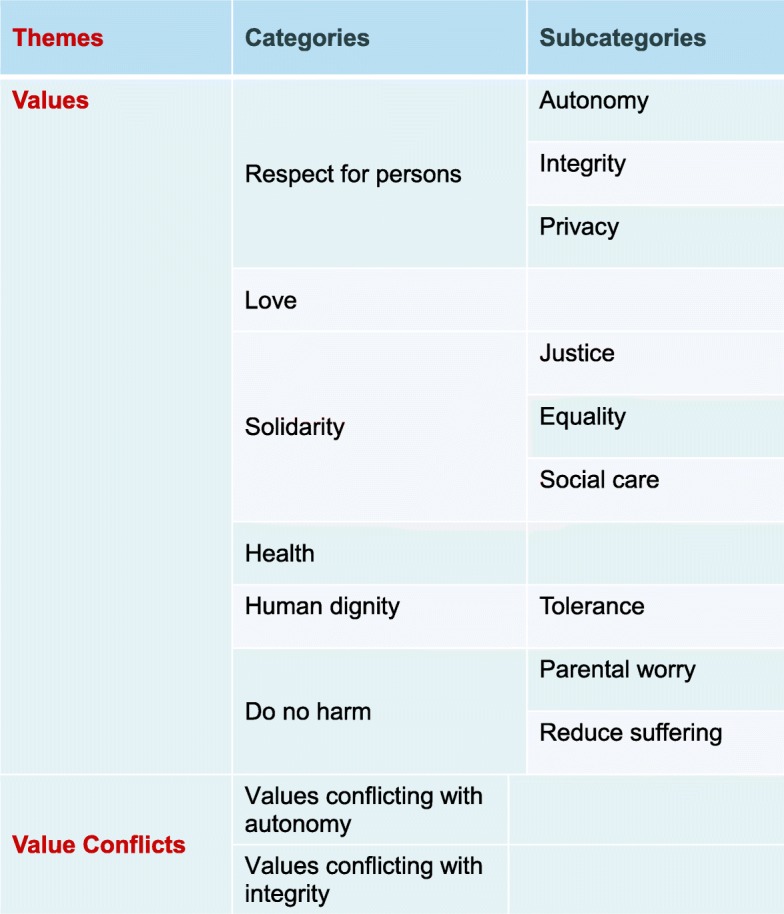
Overview of themes, categories and subcategories

Two categories transpired from the second theme; values conflicting with autonomy and values conflicting with integrity. The categories and sub categories are not mutually exclusive and some quotes can fit under one or more category or sub category. An overview of the results is presented in Fig. 1. In the following paragraphs, the results will be described and illustrated by quotes.

## Values

### Respect for persons

Respects for persons encompassed autonomy, privacy and integrity as subcategories. By far, respect for persons was the most frequently expressed value in relation to implementation and use of preconception ECS.

#### Autonomy

Participants emphasized the importance of ensuring voluntariness of participation of couples in preconception ECS and that this should not be regarded as part of routine or regulated by the state, but a true choice to make. In addition, attention should be given to both the process and the content of informed consent while monitoring of the procedure of consenting to preconception ECS.

*“How it’s actually done, interviewing people afterwards. What have they really grasped of this information? And how do they perceive this information, has it been sufficient, if it’s been over-information? I mean, if you screen for a very large number of diseases, the complicated information process… I don’t know, really, how they shall do it, but... I think that you can follow or monitor the quality of the information and, as I said, in different steps. If both partners in a partnership are carriers, what kind of information will they get? And you can see, look at the quality there.” (Physician 1).*

According to the participants, preconception ECS could be an opportunity to promote reproductive autonomy. However, some respondents voiced concerns regarding the framing and amount of information to potential parents, which may confuse rather than enlighten them.

Respondents stated that in Sweden, the Patient Act and the Patient Data Act are legal frameworks to protect patients’ autonomy and privacy, which some informants believed, paralleled with increased individualization in the society, where one’s opinions and choices were prized and encouraged.

“Internal autonomy strife”, a term used to describe conflicts arising between partners while making decisions regarding preconception ECS, was raised by participants as another challenge to parents’ reproductive autonomy.

#### Integrity

Some respondents stated that threats to one’s integrity can be precipitated by social pressure to screen and commercial interests of screening companies lobbying for use of their panels. Moreover, one respondent stated that potential parents are put in a situation where:

*“…it forces them to make choices that they ..otherwise... don’t have to make. In the position they are now, they don’t have to consider those things. But with a program like this, with the information that they would have, it would be difficult not to take that into account and that would force them to make choices that they today do not have to even think about.” (Physician 2).*

#### Privacy

Moreover, the informants voiced concerns of privacy infringement of family members and probands who did not participate in the screening program, in cases of couple’s positive results.

### Solidarity

Though respect of persons in the form of promoting autonomy and protecting integrity and privacy were the most frequently quoted values, concepts related to solidarity were alluded by all participants. The major principles reiterated by participants were concepts of *equality, justice* and *social care.*

#### Equality

The participants said that there was an emphasis in the Swedish legal framework, particularly in the context of healthcare, on equality of people, regardless of their socio- economic status, age, sexual orientations, religious affiliations, or their choice of lifestyle. For example, one informant said:

*“It’s really the view on people, perfect and non-perfect so to speak. And everybody, all should be, according to human values, be alike. I mean, it’s very, very basic... It comes with, I mean, sex, religion, whether you are LGBT person or not, that’s in the same area, so to speak.” (Political party representative).*

The example the informants presented was values founding priority setting measures. The measures were put in place to ensure, among other things, equal treatment of all who access healthcare, according to respondents.

*“….. that some things shouldn’t be taken into account because people should be treated equally. So different economic situations or different social situations or different social standings or if you’re in different age groups, that shouldn’t be relevant for whether you get access to treatment or not... or previous lifestyle.” (Bioethicist 1).*

#### Justice

The concept of justice was reflected in the replies respondents provided on questions regarding public engagement within preconception ECS, where they highlighted the importance of involving all groups in the discussion, not merely the stronger groups in the society or patients groups of spotlight diseases, such as breast cancer. Moreover, justice was a consideration in the distribution of health resources and ensuring programs such as preconception ECS is worthy of spending resources on. In addition, there was worry that the inequalities existing in the society can be imported to screening programs as preconception ECS, which may result in a further worsening of the health of disadvantaged social groups and rise of elite classes. One participant stated:

*“I’m rather skeptical towards having patient involvement, because you have to choose some patient representatives rather than others and you often choose among those who are vocal and strong enough. So those with the most serious diseases perhaps will have the weakest voice. And I think there’s a problem of justice here, so to speak.” (Bioethicist 3).*

#### Social care

Solidarity manifested in the form of delivering social care to those born with disability, was underscored by respondents as a vital current practice. According to informants, there seemed to be a lot of resources available for the disabled presently. However, the future moral outlook may change particularly if many disabled children are born, because they can prove economically expensive to take care of. Besides, it constitutes the second principle in the prioritization of health resources in Sweden and it emphasizes maximizing benefits to the most in need in the society. Moreover, society should allow for children of all sorts to exist.

*“Then, if you give birth to a child with a disorder ... the society will take care of you and your child. Just as it has always been. Perhaps with... sometimes with inferior quality, but at least it will not be stigmatizing those that prefer not to test.” (Physician 1).*

### Do no harm

This category encompassed concepts related to reduce suffering, whether that is in the form of preconception ECS inducing parental worry or reducing suffering by averting the birth of very sick children.

#### Parental worry

Most respondents iterated the potential for parental feelings of worry, anxiety, guilt and even fright if parents realized their genome was imperfect and might pass on their defective genes to their children. This may worsen their quality of life and inadvertently put them off having children. Further research to examine psychological impact of undergoing preconception ECS test on parents was requested.

*“If you get the whole genome, who wants to know all the risks for everything. I think it destroys life.” (Physician 3).*

#### Reduce suffering

The potential for preconception ECS to reduce suffering was recognized by participants, particularly in cases of diseases characterized by severe dysfunction and persistent pain. In fact, consideration of cost should always come secondary to reduce suffering, since it is morally more valuable. Severe diseases are detrimental to the affected child, parents and the family at large. The society can thrive better if there is less suffering, as explained by one informant:

*“…diseases or the painful disabledness which is very painful, that’s worth high priority. Very high. And if it’s a disease that maybe is not that painful, but you know that the children will die in two years, then it’s a very high priority. If it’s concerning the suffering, concerning the children, that’s the main question, the really main question. And the cost for treatment is not of importance, but... That’s not a priority. The suffering, the potential suffering for the children is the most important. To give birth to children that you know have a life with very much pain in one, two years and then the child will die. It’s terrible. It’s terrible for the children, it’s terrible for the parents.” (Theologian).*

### Human dignity

The category of human dignity reflects the inherent worthiness of humans and tolerance of the disabled. Respondents were of the view that respect for human dignity may be challenged with programs such as preconception ECS, because it could change the view on human value. One reason is extensive utilization of technology in reproductive medicine causing, what one respondent termed, “technifying” views on human beings and creating baby factories. Another cause could be that healthcare systems may relay a message to the public that only perfect people are encouraged to exist, when they screen for risks of defective genes and not actual diseases. There was also a fear that parents would ask to abort fetuses with less severe diseases or based on physical attributes such as brown eyes.

*“And the conflict of the human value... I guess that’s where I started. I think it’s very important that we respect sort of the human value. And not to technify the human being. That the human being is the result of all kinds of technical [interventions].” (Legal expert).*

#### Tolerance

Demoting human value may lead to intolerance to disability, as this respondent stated:

*“This selection of elite people, which I think is a dangerous way to go. It also puts intolerance and this kind of... intolerance to these people who do not fulfill these criteria if you should... you can do abortion for all these four hundred and ninety-nine conditions. That creates... I think it creates an intolerant society.” (Physician 3).*

### Health

According to informants, preconception ECS can impact the conception of healthy. One way is by redefining ill-health to encompass “at risk” conditions that needs “fixing”. Another is by changing the traditional setting of healthcare, where an individual visits her/his doctor when s/he experiences a health problem to a situation where healthcare professionals approaches healthy individuals with no prior risk to get tested.

*“[Preconception ECS] sort of puts up a standard that you are not supposed to be someone that is at risk or risks (of) putting children to this world that are suffering from disorders. And that would have impact not only on ... on these specific disorders and these... I mean, it could spread to other disorders and it could spread to the conception of poor health in general.” (Physician 2).*

### Love

Love was mentioned in two contexts. In the first case, it was described as a value that would overrule pragmatism and induce couples to remain together, instead of choosing a genetically compatible partner, in case of test positive results.

*“When it comes to love, I think that emotions overrun the... There are not so many pragmatic people around today, we don’t know in the future, but... It’s... If I may just take a one example? These patient organizations and many of them are about disorders with a distinct genetic component, say diabetes for instance. But there are very many couples that meet there, young people who meet there and then will marry, and they are fully aware of the enhanced risk for their children, of course.” (Physician 1).*

In the other context, participants mentioned research that showed that love within a couple can be threatened when they are caring for a severely ill child.

## Value conflicts

In this theme, respondents discussed how certain values can be at odds with another in the discussion of preconception ECS. At one end preconception ECS can promote a certain value, however, this could contradict with another moral value possessed by or resulting from the technology. The theme is categorized into autonomy value conflicts and integrity value conflicts.

### Autonomy value conflicts

Value conflicts may arise between promoting autonomy and cost effectiveness, as some respondents suggested. One informant stated:

*“There is this again inbuilt tension between autonomy as a value and cost-effectiveness, because if you want to make sure that you respect the autonomy of participants you would have to have rather ambitious, I would say, information for all participants. And that would be costly in itself. So... There’s sort of an inbuilt conflict between autonomy, respecting autonomy on the one hand, and cost-effectiveness on the other hand.” (Bioethicist 3).*

Another value conflict that may transpire, as asserted by other respondents, lies within autonomy itself. Since the promoted objective of preconception ECS is enhancing reproductive autonomy by presenting carrier status and disease information to potential parents, there is a risk that couples become overwhelmed by the information, causing a compromise to their autonomy. Secondly, promoting autonomy can conflict with respecting autonomy as some couples may opt out of preconception ECS and that can be interpreted as their wish to be less autonomous. Thirdly, the information ensuing from preconception ECS may restrict a person’s freedom in some ways, for example, choosing a partner only who is compatible with their DNA.

Another value conflicting with autonomy was respecting human dignity. According to the participants, the intrinsic worthiness of being a human should be respected regardless of their affliction or disability and this can be incompatible with the concept of promoting autonomy in preconception ECS, which allows to screen away some diseases and disabilities. This was explained by this quote,

*“I see the values for the individual couple... their free will to do what they want as independent couple and independent person and the value for (respecting) human life” (Physician 3).*

### Integrity value conflicts

As for values conflicting with integrity of potential parents, a utilitarian view of maximizing benefits, whether in the form of reducing disease burden or promoting science/research advancement, by such a program may clash with maintaining integrity of potential parents. In addition, autonomy of potential parents can conflict with privacy of proband family members as this quote describes,

*“There might be a conflict between these two individuals, they want to have... to take part in this (preconception ECS), but you get access to information that might be in conflict with the privacy of other people around these, as genetic relatives to these people too” (Bioethicist 1).*

## Discussion

**Fig. 2:**
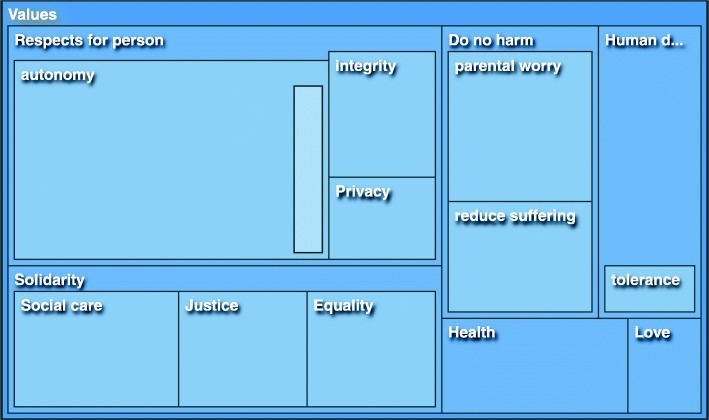
A tree map illustrating the categories and subcategories of the first theme: Values. Each dark blue rectangle denotes a category and the lighter colored rectangles within denote subcategories. Judging by the number/size of reference respect for persons and solidarity are the largest two categories

Experts in our study referred to and described particular values and principles and their conflicts when they discussed ethical and social implications of implementation and utilization of preconception ECS in Sweden. Solidarity and its subcategories of equality, justice and social care, were discussed by all experts, whereas respect of persons with reference to autonomy, integrity and privacy constituted the lion’s share of reference, even if it was not mentioned by all interviewees. It can be established that both these categories were of paramount significance to our experts in addressing preconception ECS. Tagging behind those categories are values of do no harm, human dignity, health and love, respectively. A tree map illustrating the number of quotes is found in Fig. 2. It shows how *Respect for persons* and *Solidarity* are the two largest categories.

Since Sweden is a welfare state, where its healthcare system is publicly funded, it is no surprise that values such as social care, equal value of people and justice were prominently pronounced during the interviews with the experts. In addition, respondents addressed principles of autonomy, privacy and integrity, which are endorsed by Swedish laws such as the Patient Act (SFS 2014:821) [36] and the Patient Data Act (SFS 2008:355) [37].

Human dignity seems to mean different notions to different commentators, which make its definition evasive. But the founding notion appears to be that there is an intrinsic value in being a human [32, 38], which was affirmed in the Universal Declaration on Human Rights of 1948 [39]. Other conceptions associated with human dignity are sanctity of life, non-commodification or commercialization of human beings or their organs/tissues, equality of all humans in terms of worth and rights, as well as respect of their moral agency [13, 32, 38].

Being an all-embracing concept of many notions, human dignity has been employed in debates concerning consent procedures in biobanking and data sharing, commercialization of human beings associated with patenting of genetic materials, and lastly denigration of human worth arguments in relation to embryonic stem cell research and reproductive cloning [32]. In our study, participants referred to human dignity when they mentioned the threat to inherent value of human beings as result of “technifying” them, or turning offspring into products, or sorting humans into perfect or non-perfect ones, or permitting only certain types of humans to exist. In another context, participants mentioned human dignity in connection to equal treatment of patients within Swedish healthcare, regardless of their religion, socioeconomic status, gender, age and lifestyle. In short, participants used the non-commodification and equality of humans arguments in discussing implementation and use of preconception ECS.

If human dignity proved complicated to define, elucidating solidarity is as problematic. Prainsack and Buyx [31] identified four distinctive contexts in which solidarity emerged within bioethics. One setting occurs when validating governments’ interference for promoting collective good over individual benefit within public health system. Secondly, as a justification or its lack thereof, for delivering aid to poorer countries within a global health setting. The other two contexts where solidarity is brought up are relevant to our study; the first of which is within welfare states for just and equitable allocation of resources within healthcare system and social care. In this setting, solidarity is further characterized in terms of risks, income and lifestyle and associated with concepts of justice and equity [31]. This was pinpointed by interviewees’ replies when they referred to non-discriminatory dispensing of healthcare to patients regardless of their choice of lifestyle or socio economic status. The results show that solidarity can be further characterized in terms of genetic defects or handicap, not merely risks, lifestyle and income. Lastly, solidarity was described by Häyry [12] as a distinctively European value, together with dignity and precaution, contrasting to American liberal values of autonomy, beneficence and justice. Further, solidarity becomes one of the founding values for European Union healthcare systems, of which Sweden is a part [14].

Prainsack and Buyx [31] define solidarity as “shared practices reflecting a collective commitment to carry costs to assist others”. The “costs” can be categorized as, for example, economical, emotional or societal. Furthermore, solidarity can be enacted between individuals, or at a group level as a social norm, or lastly institutionalized into a legal system. The latter is characteristic of welfare states with taxpayers’ financed healthcare systems, such as Sweden. Concepts associated with solidarity, such as social care, justice and equitable use of resources were prominent in participants’ responses. Furthermore, they referred to contractual level of solidarity in Sweden, as delineated in priority setting measures [15].

The World Health Organization (WHO) defined health as the status of an individual with “complete physical, mental and social well-being”, and not simply as lack of disease or functionality [40]. Furthermore, possessing the best obtainable health is viewed as a human right, according to the WHO [41]. Philosophers adopted naturalistic or evaluative approaches to define health. Though both concurred that health is lack of ailment or pathology, evaluative theorists stated that disease is influenced by temporal assessment of notions, such as righteousness, well-being and perfection [42]. The naturalists, on the other hand, defines pathology with reference to the biostatistical theory, where normal ranges of function of body processes and organs are constructed. Beyond these normal ranges lie disease [42].

Health, as discussed by respondents, incorporated concepts of well-being and quality of life. But they also acknowledged that parents may define ill health, or disability subjectively, influenced by their cultural, educational and religious bearings. Furthermore, there were contemplations, whether carrier screening programs that identify carrier status of defective genes in a healthy population can redefine the notion of ill health.

With reference to value theory, health is considered a value both intrinsically and extrinsically. Health in itself and for its own sake has a value, so being healthy has its own worthiness, but it is also instrumental for one’s well-being, access to opportunity, exercise of autonomy and other capabilities [42].

Respects for persons was first featured in Belmont Report of 1979, as one of the founding values for participation in research. The main manner this can be professed is via respecting autonomy and safeguarding those with diminished capacity for autonomy [43]. Later, autonomy was embraced as the first principle in biomedical ethics by Beauchamp and Childress [11] and incorporated in European bioethics and bio-laws [13, 38]. According to the later, autonomy is multi-faceted and include; capability of self-determination, moral cognizance, accountability and political participation, informed consent and undertaking decisions and acting upon them with no external hindrance [13, 38].

To our participants, autonomy is a substantive principle to respect in implementation and use of preconception ECS, from ensuring voluntary participation of potential parents without external pressure, to circumventing routinization of the screening program as a threat to autonomy, to ensuring proper informed consent procedures and delivering of information, to discussing amount and quality of information given by healthcare professionals. Furthermore, experts respected parental pluralistic moral outlooks and the ensuing decisions. Therefore, respondents addressed most, if not all, of the aspects of autonomy as detailed in the European bio-laws. One addition the experts divulged was internal autonomy strife, occurring because of differing views between partners concerning participation in a preconception ECS program.

In addition to autonomy, Johnsson and Eriksson [30] added the principles of integrity and authority as essential components in respect of persons. Integrity is the principle that grapples with one’s social and personal realms. Respecting integrity means acknowledging and withdrawing from a person’s personal, psychological and physical spaces unless invited into them [30]. Preconception ECS, experts expressed, may threaten potential parents’ integrity. In their description, integrity was a parent’s personal space, that could be infringed upon by social pressure to test or the lobbying of commercial companies to screen or healthcare professionals’ offer of screening tests to couples. The prospective parents would thereby be compelled to undertake decisions that they would, otherwise, have not known existed.

Lastly, preconception ECS can precipitate value conflicts, which respondents highlighted in respect to autonomy and integrity. They discussed risks of encroaching on the privacy of genetic relatives while promoting reproductive autonomy of couples; advancing commercial interests and intruding on couple’s integrity by offering the test; promoting reproductive autonomy while respecting human dignity are some example of potential value conflicts. Furthermore, the new HT could initiate conflicts with existing societal values, such as equality or solidarity, because it can induce a form of elitism. Hofmann [5] discussed how new HTs are value-laden and could trigger conflicts with societal values, which hinder their dissemination. Assessment of ethical and social values as part of HTA was proposed as a way to circumvent the problem of HT diffusion. This could be achieved by identifying such value conflicts and addressing them or by anticipating, in advance, if a new HT will be accepted by the society or not.

## Conclusions

Our study examined values and value conflicts as reiterated by health policymaking experts in Sweden, when they described ethical and social implications in implementation and use of preconception ECS. The analysis of the interviews disclosed that respect for persons, solidarity, human dignity, do no harm, health and love were the main values inferred to by experts. In addition, they discussed value conflicting with autonomy and integrity, and, for instance, priority setting and human dignity. Moreover, the analysis revealed that certain values were deemed more substantive than others, judging by the extent and detail of inference; for example, respect for persons and solidarity were on top of the list.

Hence, it can be concluded that the interviewed experts highlighted values and concepts that are distinctive of welfare states such as Sweden and that preconception ECS could challenge such values. Determining these conflicts could prove useful to circumvent a dissemination problem, in case of implementing the new technology. Though our analysis relied on a small sized sample, we believe the results are pertinent to countries with similar contexts.
